# Unveiling the unsealed: modified Zenker’s peroral endoscopic myotomy with open incision

**DOI:** 10.1016/j.igie.2024.04.004

**Published:** 2024-04-09

**Authors:** Farimah Fayyaz, Eric Swei, Mouen Khashab

**Affiliations:** Johns Hopkins Medicine, Baltimore, Maryland, USA

Peroral endoscopic myotomy for Zenker’s diverticulum (Z-POEM) has demonstrated safety and efficacy with high rates of technical and clinical success.^1^ However, the technique is associated with certain drawbacks, such as the technical difficulty in mucosal closure, which contributes to prolonged procedural duration and increased costs, and the resulting remnant mucosal flap, linked to clinical failure and recurrence. To overcome these limitations, here we introduce a novel technique called open Z-POEM.

## Case presentation and endoscopic technique

**Figure 1:**
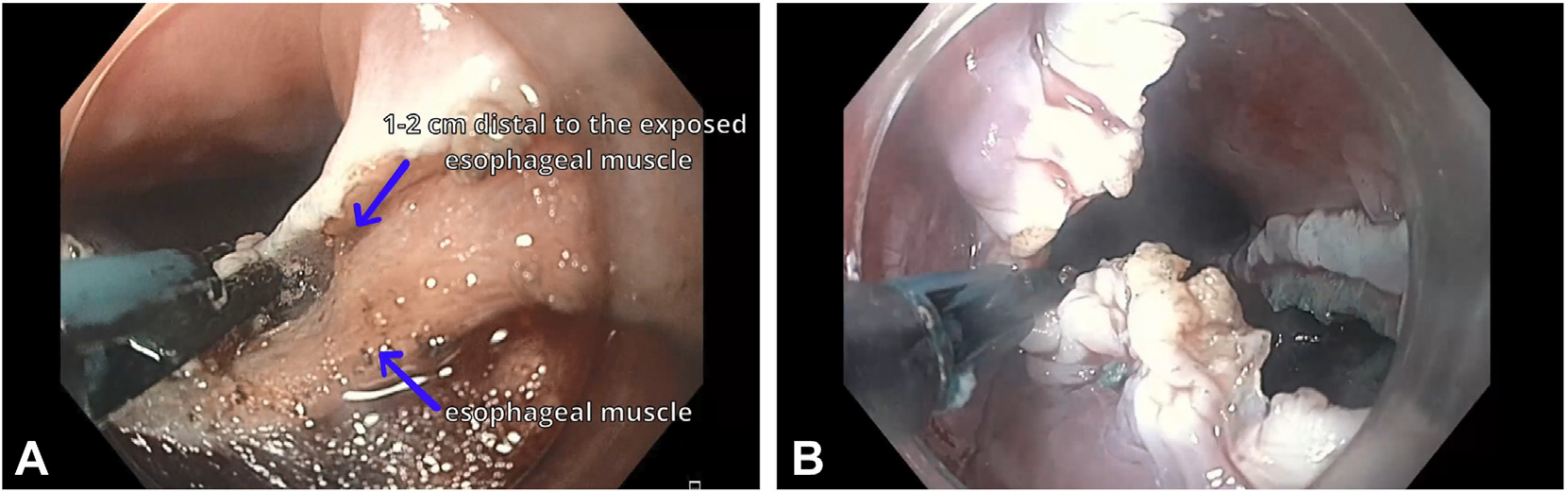
Mucosotomy in open Zenker’s peroral endoscopic myotomy. **A,** Distally on the esophageal side to just below the exposed esophageal muscle. **B,** Proximally from the initial mucosal incision to the base of the diverticulum.

**Figure 2:**
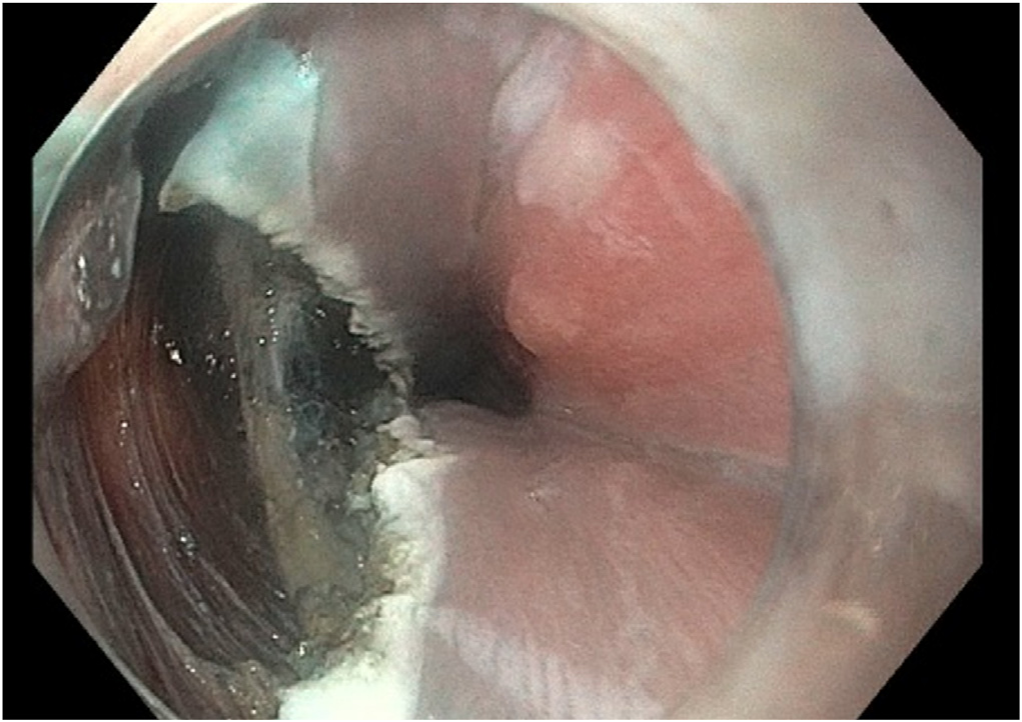
Leaving the defect open in open Zenker’s peroral endoscopic myotomy.

For the open Z-POEM technique (Video 1, available online at www.igiejournal.org), an over-the-septum submucosal injection is performed, followed by mucosal incision and submucosal tunneling to fully expose the septal muscular layer. Subsequently, as per the standard Z-POEM technique, a complete myotomy of both the diverticular and esophageal muscles is carried out up to the base of the prior Zenker’s diverticulum, ensuring the thorough transection of the cricopharyngeal muscle, and extending a few centimeters into the esophageal muscle.^2^ Afterward, to maximize the communication between the diverticulum and esophageal lumen and to prevent any residual pouches, a mucosotomy is performed on both sides of the incision to just below the septum to enhance the passage of food (Fig. 1). Finally, the defect is left open, and no clips are used for incision site closure (Fig. 2).

This novel technique was performed by a single operator (M.K.) on 24 consecutive patients (11 women; mean age, 76.3 ± 11.3 years) diagnosed with Zenker’s diverticulum, achieving 100% technical and clinical success at the 1-month follow-up (Table 1). No endoscopic clips were used, and the mean procedure duration was 19.3 ± 6.8 minutes. No intraprocedural adverse events occurred.

**Table 1:** Baseline characteristics, procedural characteristics, and clinical outcomes of patients undergoing open Zenker’s peroral endoscopic myotomy

Characteristics and outcomes	Values
Baseline characteristics	
Female sex	11 (45.8)
Age, y	76.3 ± 11.2
Diverticulum size, mm	29.1 ± 17.9
Procedural characteristics	
No. of endoscopic clips used	0 ± 0
Hemostatic gel application	19 (79.2)
Procedure duration, min	19.3 ± 6.8
Technical success	24 (100)
Clinical outcomes	
Hospital length of stay, days	2 (1-2.25)
Postprocedural adverse events, mild/moderate/severe	0/1 (4.2)/1 (4.2)
Kothari-Haber score	
Preprocedure	5 (3-8)
Last follow-up	0 (0-1)
Follow-up period, wk	4 (1-24.5)
Clinical success∗	24 (100)

Values are mean ± standard deviation, n (%), or median (interquartile range).

∗ Clinical success was defined by a postprocedural Kothari-Haber score of <3.

Postoperatively, 1 moderate adverse event occurred in a patient (4.2%) who experienced recurrent dysphagia 3 weeks after the procedure. EGD showed an esophageal ulcer, which was managed conservatively with outpatient medical therapy, resulting in complete resolution of symptoms. In addition, 1 severe adverse event (4.2%) was observed in a patient with a history of obstructive sleep apnea managed with continuous positive airway pressure. The postprocedure esophagography revealed an esophageal leak, which was successfully treated with endoscopic vacuum therapy. The patient’s dysphagia symptoms had completely resolved by the last follow-up.

Notably, the procedure has undergone refinements and enhancements over time. These refinements include using a scissor-type knife for all aspects of the procedure, from mucosal incision to the final mucosotomy, and application of hemostatic gel at the defect site to promote healing and prevent bleeding.

## Discussion

Given that the mucosa is left intact in standard Z-POEM, a substantial mucosal flap might lead to persistent symptoms.^2^ Open Z-POEM represents a modified approach addressing the challenges of mucosal closure and mucosal flaps by effectively removing the remnant mucosal flaps and leaving the defect open.

In large diverticula, food stasis can cause submucosal fibrosis and adhesion of mucosa to the muscle, potentially resulting in incomplete myotomy. To address this, the addition of the mucosotomy step allows the simultaneous incision of residual muscle tissue adhered to the mucosa. Additionally, leaving the defect open permits the extension of mucosotomy and removal of any mucosal flaps and potentially decreases the recurrence rate. Because submucosal tunneling is performed in this technique and the endoscopist is able to control any additional injury, the risk of perforation is minimal.

The open Z-POEM technique, demonstrated in our case series, yielded a mean procedure time of 19.3 ± 6.8 minutes. In comparison, an international multicenter study on standard Z-POEM reported a mean time of 43.8 ± 19.2 minutes.^3^ Despite our study's limitations (single operator and small sample size), our result suggests a potential reduction in procedure time with the open Z-POEM technique. Prospective comparative studies are warranted to further validate these observations.

In conclusion, open Z-POEM represents a modified and simplified Z-POEM approach. This method involves mucosotomy and remnant flap removal to potentially reduce recurrence rates. By leaving the defect open, this technique eliminates the challenges associated with mucosal closure, resulting in reduced procedure time and cost.

## Patient consent

This was a retrospective study, and the IRB permitted the research to be exempt from obtaining informed consent.

## Disclosure

The following author disclosed financial relationships: M. Khashab: Consultant for Boston Scientific, Medtronic, Olympus America, Pentax, GI Supply, Apollo Endosurgery, and Triton; royalties from UpToDate and Elsevier. All other authors disclosed no financial relationships.

## Supplementary data

Video 1 Open Zenker’s peroral endoscopic myotomy: a modified Zenker’s peroral endoscopic myotomy with an open incision.
